# An Investigation of Fatty Acids, Crude Protein and Total Solids in Colostrum in Relation to Health Status Regarding Postpartum Dysgalactia Syndrome and Certain Parity Groups of Sows

**DOI:** 10.1111/jpn.70100

**Published:** 2026-08-06

**Authors:** Stephan Rosengart, Florian Lohkamp, Bussarakam Chuppava, Hubert Henne, Anne Kathrin Appel, Lea‐Sophie Trost, Jens Tetens, Imke Traulsen, Ansgar Deermann, Michael Wendt, Christian Visscher

**Affiliations:** ^1^ Institute for Animal Nutrition University of Veterinary Medicine Hannover Hannover Germany; ^2^ Clinic for Swine and Small Ruminants, Forensic Medicine and Ambulatory Service University of Veterinary Medicine Hannover Hannover Germany; ^3^ BHZP GmbH Dahlenburg‐Ellringen Germany; ^4^ Department of Animal Sciences, Livestock Systems University of Göttingen Göttingen Germany; ^5^ Department of Animal Sciences, Functional Breeding University of Göttingen Göttingen Germany; ^6^ Center of Integrated Breeding Research University of Göttingen Göttingen Germany; ^7^ Institute of Animal Breeding and Husbandry Kiel University (CAU) Kiel Germany; ^8^ EVH Select GmbH Meppen Germany

**Keywords:** colostrum, fatty acids, parity group, postpartum dysgalactia syndrome, sow

## Abstract

Colostrum is the main source of nutrients and proteins for newborn piglets. To give each piglet the best possible start in life, it is necessary to evaluate the colostrum according to quality. This has to be done by analysing the components, as component composition is a main quality parameter. To achieve this aim, in this study, the content of total solids, crude protein and 24 fatty acids in colostrum samples was analysed. The colostrum samples from 334 sows were classified according to the parity of the donor sow. Parity group 1 contained parity 1 and 2 sows (*n *= 93), parity group 2 contained parity 3 and 4 sows (*n* = 109), parity group 3 contained parity 5 and 6 sows (*n* = 61) and parity group 4 contained sows in parity 7 and higher (*n* = 71). In addition, 187 sows were investigated clinically within 2 days after farrowing and divided into three groups according to their health status with regard to postpartum dysgalactia syndrome (PDS) (healthy [*n* = 83], clinically suspicious [*n* = 74] and diseased sows [*n* = 30]). In this way, it was tested whether puerperal diseases affected the content of fatty acids, crude protein and total solids. Only a few of the colostrum constituents were directly linked to the health of the sows regarding PDS after farrowing. When comparing the contents of the different fatty acids in % of total fatty acids, the content of C18:0 in the colostrum of clinically suspicious sows was higher than in the colostrum of sows without PDS. The colostrum of sows without PDS had a higher content of C18:2n6c and C18:3n3 than the colostrum of clinically suspicious sows. Furthermore, the colostrum of PDS‐diseased sows had a higher proportion of C20:2 than that of clinically suspicious sows. With regard to C24:1, the colostrum of sows without PDS had a higher proportion than that of PDS‐diseased sows. In contrast, the parity group of the sows affected the quantity of 15 of the 24 analysed fatty acids. With regard to the proportion of the colostrum constituents in the whole colostrum, the colostrum of younger sows up to the 4th parity compared to older sows often had higher proportions of fatty acids (16 out of 24 parameters with significant differences), especially n‐3 (C20:5n3, C22:6n3, C18:3n3) compared with n‐6 (C18:2n6c, C18:3n6, C20:3n6, C20:4n6) and n3:n6 ratio (parity group 1: 14.6; parity group 2: 15.1; parity group 3: 15.4 and parity group 4: 15.5). No differences in total solids or crude protein were observed between the health groups regarding PDS or among the parity groups. In summary, there was rarely any link between health status regarding PDS and colostrum constituents, as opposed to a more consistent link between parity groups and colostrum constituents.

## 1 Introduction

Colostrum is a rich natural source of macro‐ and micro‐nutrients and immunoglobulins (Csapó et al. 1996) and is the main source of newborn piglets for nutrients and peptides, contributing strongly to passive immunity (Widdowson et al. 1976; Zhang et al. 1998; Theil et al. 2014; Farmer et al. 2019). In addition, high colostrum intake is associated with low piglet mortality during the suckling period, a high daily weight gain until weaning and a high piglet body weight at weaning (Decaluwé et al. 2014; Declerck et al. 2016). Up to now, it is known that colostrum synthesis starts about 10 days before farrowing and ends a few hours after farrowing (Palombo et al. 2018; Quesnel and Farmer 2019). PDS in sows adversely affects both the litter weight at weaning and the daily weight gain of their piglets (Mirko and Bilkei 2004; Sărăndan et al. 2009; Studer et al. 2024). In addition, PDS in sows is often accompanied by fever and endometritis (Angjelovski et al. 2019). The detailed effects of PDS on sows are poorly understood. There is evidence that, in cases of PDS, previously milked colostrum contains higher lactose concentrations and lower protein and sodium levels (Gooneratne et al. 1982). Potential causal factors may include a prematurely initiated milk production process and early damage to individual cells (Gooneratne et al. 1982). In addition, a previous study showed that abdominal mammary complexes are more commonly affected by mastitis compared with pectoral mammary complexes (Baer and Bilkei 2005). What is clear, however, is that the increasing number of piglets per litter in recent years has exacerbated the challenge to supply all piglets with sufficient amounts of colostrum (Moreira et al. 2017; Kobek‐Kjeldager et al. 2020). Colostrum yield tended to be highest in parity 2 and 3 sows (Devillers et al. 2007). Another previous study reported that parity does not significantly influence colostrum yield, at least up to parity 9 (Quesnel 2011). Thus, it is likely that not only the amount of ingested colostrum but also the individual composition of ingested colostrum has an influence on the performance and health of suckling piglets. Therefore, it is necessary to achieve the best possible quality of colostrum, especially in litters from hyperprolific sows. The fat content in colostrum exhibits the greatest variability (Hurley 2015). The physiological function of fatty acids in colostrum is poorly understood up to now (Metzler‐Zebeli 2021). It is known from cell culture studies and rodent models that some n‐3 polyunsaturated fatty acids (PUFA), such as C22:6n3, trigger anti‐allergic and anti‐inflammatory immune responses and n‐6 PUFAs, such as C20:4n6, trigger pro‐inflammatory immune responses (Belluzzi 2002; Pearl et al. 2014; Zárate et al. 2017). A low n‐6:n‐3 ratio (3:1) tends to decrease the number of piglet losses 14 and 21 days after farrowing compared with a higher n‐6:n‐3 ratio (9:1 and 13:1), but the number of liveborn piglets tends not to be different (Yao et al. 2012). So far, there are several studies that have shown that the fatty acid pattern in the sow's diet during late gestation and lactation has an influence on the fatty acid pattern in the colostrum and in the milk (de Quelen et al. 2013; Bai et al. 2017; Jang et al. 2020; Nguyen et al. 2020). For example, a low n‐6:n‐3 ratio (4:1 compared with 13:1 and 10:1) in the feed is transferred to the colostrum (8:1 compared with 12:1) (Nguyen et al. 2020). In addition, a high level of extruded linseed (rich in n‐3 fatty acids) in the diet of sows leads to a higher level of C18:3n3 in the milk compared with the milk of sows fed with a high sunflower oil level in the diet (de Quelen et al. 2013). This means that the fatty acid content in the colostrum can be influenced by the sow's diet and affects the intake by suckling piglets. This study has a special focus on fatty acid contents in colostrum, as it can be influenced by the feed. In addition, there is also evidence from piglets that the fatty acid content of feed influences the mRNA expression of claudin‐1 and zona occludens 1, which are indicators of the intestinal epithelial barrier in the intestine (Chen et al. 2019). Thereby, n‐3 fatty acids appear to have immunomodulatory effects in rodents (proliferation of lymphocytes and natural killer cell activity) (Peterson et al. 1998). In addition, Chen et al. 2019 reported an increase in interleukin‐10 in the mucosa of the jejunum of suckling piglets from sows that were fed more n‐3 fatty acids (added 68.2 g coated n‐3 PUFA/kg basal diet). Leonard et al. 2010 detected a greater percentage of phagocytic activity of leucocytes and lymphocytes in piglets’ blood in suckling sows that were fed a fish oil supplement as a source of n‐3 fatty acids. Furthermore, there is a negative correlation between the parity of a sow and the levels of protein, solids‐not‐fat and the density in its transient milk sampled from 36 to 72 h postpartum (Bondoc et al. 2020). Another study reported more lactose and N‐acetylglucosamine and less IgG in the colostrum of gilts compared to the colostrum of sows in second, third and fourth parities (Luise et al. 2020). Also, a previous study reported more fat and a higher somatic cell count in the colostrum of gilts compared to the colostrum of older sows (Amatucci et al. 2022). The literature shows that the content of many colostrum constituents is influenced by the sows’ parity. However, to date, there are no studies on the extent to which the content of individual fatty acids in colostrum is associated with the puerperal health status regarding PDS and the parity of sows. The knowledge about the relation between the puerperal health status regarding PDS and parity and the content of fatty acids, crude protein and total solids of colostrum can help to adjust cross‐fostering closer to the performance potential of each individual sow and a more precise fatty acid‐adapted feeding.

Therefore, the aim of this study was to investigate the influence of postpartum dysgalactia syndrome (PDS) and the parity group of sows on fatty acids, crude protein and total solids of colostrum.

## 2 Materials and Methods

### 2.1 Animals, Housing and Feeding

The study took place on a farrow‐to‐feeder farm in Lower Saxony, North Germany, from August 2019 to February 2021. All sows were housed under the same environmental conditions, including an automated system to control the stall temperature. The farm kept about 500 hybrid sows (db.Viktoria, BHZP Large White × BHZP Landrace, German Federal Hybrid Breeding Programme [BHZP GmbH, Ellringen, Germany]) and batch farrowing was carried out every 2 weeks. The barn consisted of four farrowing units with 24 farrowing pens (ProDromi, Vereijken, Ede, the Netherlands) each. Twelve pens were located on each side of the working aisle. The farrowing units were forcibly ventilated by a barn exhaust fan in the middle and a porous ceiling system. None of the sows sampled had been treated with antibiotics within 7 days prior to sampling, and parturition was not induced medically in sows.

On the 109th day of gestation, 7 days before the calculated date of parturition (day −7), the sows were moved to the farrowing pen and were kept in farrowing crates until weaning. The sows were fed twice daily with a gestation diet from day −7 to day −3 (about 3750 g per day). From day −2 to day 0, sows were fed once a day with a mix of gestation and lactation diets (gestation diet about 1875 g at day −2, about 1125 g at day −1 and about 375 g at day 0, each mixed with lactation diet 0 g at day −2, about 1125 g at day −1 and about 375 g at day 0). From day 1 to day 2, animals were fed twice a day with the lactation diet (about 3750 g per day) and from day 3 until weaning three times a day with a lactation diet (d3–d5 about 5625 g per day, and d6 until weaning 7230 g). The level of performance on the farm was high (total born piglets: parity 1 healthy: 17.6 ± 2.8, parity 2 healthy: 17.4 ± 2.9, parity 3–7 healthy: 17.8 ± 3.4, parity 8 and higher healthy: 14.9 ± 3.2, parity 1 clinically suspicious: 17.2 ± 3.5, parity 2 clinically suspicious: 17.3 ± 3.2, parity 3–7 clinically suspicious: 18.0 ± 3.25, parity 8 and higher clinically suspicious: 16.8 ± 2.8, parity 1 diseased: 16.4 ± 3.8, parity 2 diseased: 16.6 ± 3.3, parity 3–7 diseased: 16.6 ± 3.7 and parity 8 and higher diseased: 14.5 ± 4.2), as published in a previous article (Rosengart et al. 2022). The nutrient content of the sow diets was analysed, the results of which are shown in Table 1 and Table S1. The chemical composition of the diet was analysed using the methods of the Association of German Agricultural Analytical and Research Institutes e. V. (VDLUFA) (Naumann and Bassler 2012). The feed was designed in accordance with the recommendations of the Society for Nutritional Physiology (GfE) (Ernährungsphysiologie 2006). The farrowing period lasted from day −3 to day 1. Feeding on the farm was highly standardised during the peripartum period; therefore, each sow, including the gilts, received the same amount of feed. This allowed us to calculate the daily feed and nutrient intake of the sows. Calculated data, including fatty acid intake, are shown in Table S2. Each animal had unlimited access to tap water.

**Table 1:** Different nutrient contents, including 25 fatty acids in the gestation and lactation diet (pelleted complete feed) in accordance with the analysis in g/kg as fed (mean ± SD).

Item	Gestation diet	Lactation diet
Total solids	887 ± 7.5	889 ± 6.9
Crude protein	142 ± 3.4	163 ± 10.1
Crude fat	30 ± 1.5	36 ± 4.0
Crude fibre	72 ± 5.8	48 ± 3.7
Crude ash	53 ± 1.4	52 ± 3.0
Calcium	7.2 ± 1.0	8.1 ± 1.0
Phosphorus	4.8 ± 0.4	6.0 ± 0.2
Energy (MJ ME/kg)	11.7 ± 0.3	13.0 ± 0.3
C12:0	0.09 ± 0	< 0.05
C14:0	0.08 ± 0.02	0.05 ± 0.005
C15:0	< 0.05	< 0.05
C16:0	5.99 ± 0.18	6.06 ± 0.34
C16:1	0.06 ± 0.006	0.06 ± 0.006
C17:0	< 0.05	< 0.05
C18:0	0.50 ± 0.008	0.70 ± 0.04
C18:1n9t	< 0.05	< 0.05
C18:1n9c	4.61 ± 0.38	4.57 ± 0.36
C18:2n6c	15.0 ± 0.42	18.47 ± 1.08
C20:0	0.07 ± 0	0.07 ± 0.006
C18:3n6	< 0.05	< 0.05
C20:1	0.17 ± 0.01	0.16 ± 0.01
C18:3n3	1.21 ± 1.2	1.82 ± 1.13
C20:2	0.05 ± 0	0.05 ± 0.002
C22:0	0.09 ± 0.002	0.09 ± 0.004
C20:3n6	< 0.05	< 0.05
C22:1n9	< 0.05	< 0.05
C20:3n3	< 0.05	< 0.05
C20:4n6	< 0.05	< 0.05
C22:2	< 0.05	< 0.05
C24:0	0.10 ± 0.02	0.08 ± 0.01
C20:5n3	< 0.05	< 0.05
C24:1	0.06 ± 0.002	0.05
C22:6n3	< 0.05	< 0.05
Total fatty acids	27.99 ± 0.53	32.19 ± 1.91
n‐6:n‐3 ratio	12.5 ± 0.36	10.18 ± 0.48

### 2.2 Sampling

For the study, a single colostrum sample was taken from 348 sows out of 41 farrowing groups (5–10 sows in each farrowing group). The sows were randomly selected. Colostrum samples were taken from the first two teats in most of the sows. In a few sows, where the milk amount was insufficient, the next posterior teats were sampled. The samples were collected close to farrowing and no more than 6 h after the onset of parturition. When three to four piglets had been born and had started suckling colostrum at the mammary gland, colostrum was collected. The last criterion was the most important one because the exact beginning of farrowing was not always known. Care was taken to ensure that at least two piglets were still wet from birth, indicating that no more than 6 h had passed since the onset of farrowing. The exact time was not recorded. The mammary glands were cleaned dry and massaged before milking started (10–20 mL, sample tubes: Schraubröhre, 30 mL, 84 × 30 mm, Sarstedt AG & Co. KG, Nümbrecht, Germany). Immediately afterwards, the samples were marked individually with sow number and date and frozen at −20°C. Sample analysis took place about 18 months after collection. The parity of the sows was registered through access to the management software db.planer (version 1906, BHZP GmbH, Ellringen, Germany). In addition, parity groups were built in accordance with a previous study (Vötterl et al. 2024). Parity group 1 contained parity 1 and 2 sows (*n* = 93), parity group 2 contained parity 3 and 4 sows (*n* = 109), parity group 3 contained parity 5 and 6 sows (*n* = 61) and parity group 4 contained sows in parity 7 and higher (*n* = 71). Furthermore, 187 of the sampled sows were clinically examined with regard to the PDS as described by Rosengart et al. (2021). For 14 of these 187 sows, the parity was unknown. Most of the 187 sows were investigated shortly after farrowing (within 24 h postpartum (same day as colostrum sampling)), and only a few sows were examined later (within 48 h postpartum). The mammary gland complexes were inspected, palpated and scored (0 = loose consistency, 1 = elastic consistency, 2 = solid consistency). In addition, the vulva was checked for purulent discharge (indication for metritis), the feed intake was checked regularly and the sow's standing‐up behaviour was assessed (diseased sows eat poorly or not at all and are often difficult to get to stand). At the end of the clinical examination, body temperature was measured with a Veterinary Thermometer VT 1831 (Microlife AG, Widnau, Switzerland). Although fever alone is not conclusive in confirming the diagnosis of PDS, it is the most important indicator. Therefore, rectal temperature was scored highest for the health score. The litters were scored on average on the fifth day of life (±1.43) using a modified score in the same way as described by Rosengart et al. (2021) (piglet score (piglets with fresh, bloody or encrusted injuries with a diameter of more than 0.5 cm on the carpal joints): 0 = no piglets, 1 = less than 50% of the litter, 2 = more than 50% of the litter). Injuries develop due to the intense rubbing of the piglets against the ground because of insufficient milk supply. All clinical data were aggregated to a health score (Figure 1 [adapted from Rosengart et al. (2022]). One point was allotted each if the sow showed no feed intake, reluctance to stand up, purulent discharge and more than two mammary gland complexes with consistency 1. In addition, one point was allotted for a piglet score of 1 and every mammary gland complex with consistency 2. Two points were allotted each for a piglet score of 2 and a rectal temperature ranging from 39.0°C to 39.4°C. Four points were allotted for a rectal temperature ranging from 39.5°C to 39.8°C. Five points were allotted for a rectal temperature higher than 39.8°C. In total, 0 to 2 points meant ‘healthy sow,’ 3 to 5 points meant ‘clinically suspicious sow’ and more than 5 points meant ‘diseased sow’. Clinical data could only be collected from sows of 26 farrowing groups.

**Figure 1:**
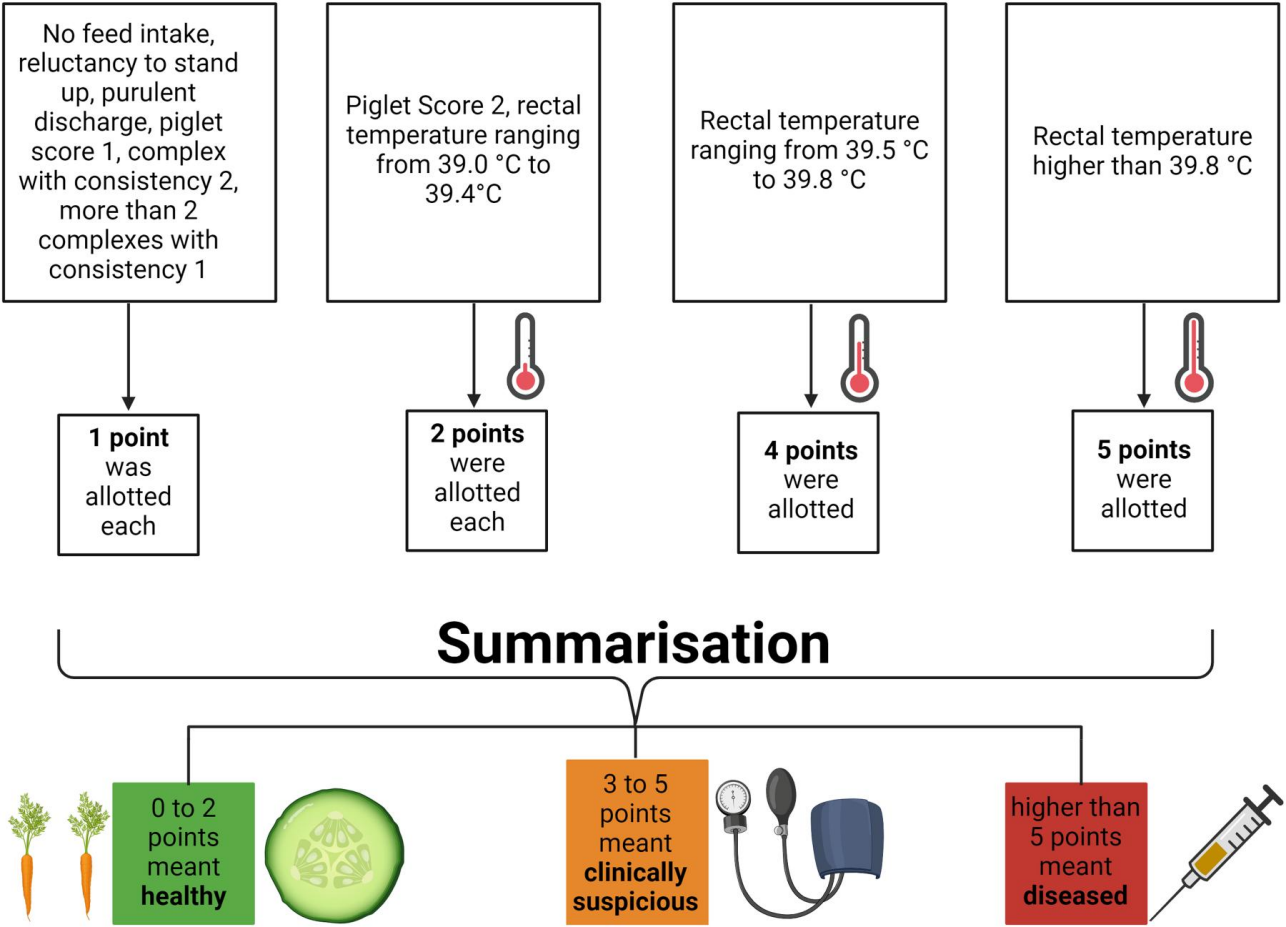
Schematic diagram on aggregation of clinical data (figure was created with BioRender.com). Evaluation: One point was assigned if the sow exhibited no feed intake, reluctance to stand up, purulent discharge, or more than two udder complexes with elastic consistency (consistency 1). Furthermore, one point was assigned for each udder complex classified as solid consistency (consistency 2) and for a piglet score of 1 (piglet score: piglets with fresh, bloody, or encrusted injuries larger than 0.5 cm in diameter on the carpal joints; 0 = no piglets affected, 1 = less than 50% of the litter affected, 2 = more than 50% of the litter affected). Two points were assigned for a piglet score of 2 and for a rectal temperature between 39.0°C and 39.4°C. Four points were assigned for a rectal temperature between 39.5°C and 39.8°C, while five points were assigned for a rectal temperature above 39.8°C. Overall, a total score of 0 to 2 points classified the sow as ‘healthy’, 3–5 points as ‘clinically suspicious’, and more than 5 points as ‘diseased’. [Color figure can be viewed at wileyonlinelibrary.com]

### 2.3 Analysis of Colostrum

The content of fatty acids in the colostrum was determined by a modified method according to Lepage and Roy (1986). Colostrum (100 mg) was poured into a narrow glass tube. The internal standard was a 2‐mL methanol‐hexane‐tridecanoic acid (C13) mixture, which was mixed with every colostrum sample. Acetyl chloride (200 µL) was added and incubated for 1 h. Afterwards, 5 mL of potassium carbonate chloride solution was added. Fatty acids were chromatographed as methyl esters on a 100‐m fused silica column with an internal diameter of 0.25 mm (Restek GmbH, Bad Homburg, Germany). The column was wall‐coated with 0.20 mm SP‐2330. Analysis was performed on a gas chromatograph Trace 1300 (Thermo Fisher Scientific GmbH, Dreieich, Germany) equipped with a flame ionisation detector. Nitrogen was used as a carrier gas. The split ratio was 67:1. The temperature of the injection port was 280°C, and of the detector 260°C. The column temperature was kept at 50°C for 2 min and, in a step‐wise (30°C/min.) fashion, reached a plateau of 120°C. A temperature of 120°C was kept for 1 min. Afterwards, the temperature was increased step‐wise at a rate of 3.5°C per min. until reaching a plateau of 240°C. A temperature of 240°C was kept for 12 min. The total amount of fatty acids was calculated by adding up the results of the individual fatty acids. The determination limit was 0.05 g/kg for fatty acids and 20 g/kg for crude protein.

Crude protein concentration was determined as nitrogen using the Dumas method, and the crude protein content was calculated (according to VDLUFA). The results of the analysis were in the range described in the literature (Hurley 2015; Metzler‐Zebeli 2021). The content of total solids was determined by drying the samples overnight in a drying oven at 103°C. All analytical measurements were performed in duplicates. Measures of analytical precision are provided in Table S3.

## 3 Statistical Analysis

The data analysis was performed using the SAS Enterprise Guide (version 7.1, SAS Institute Inc., Cary, NC, USA). Least square mean or mean, and the standard error were calculated for all parameters in g/kg as fed (Table 1), per cent of total fatty acid (Tables 2, 4 and 5) and g/kg whole colostrum (Table 3). Differences between health categories and parity groups were examined by using a one‐way analysis of variance (ANOVA), including the calculation of the error variance. For multiple pairwise means comparison between the three health categories and the four parity groups, the Ryan–Einot–Gabriel–Welsch multiple range test was used. Interaction between health and parity was determined by two‐way ANOVA. *p* values less than 0.05 were considered significant.

**Table 2:** Comparison of contents of different fatty acids in colostrum of healthy, clinically suspicious and diseased sows. Data in % of total fatty acids (LSMean ± SE).

Item	Healthy	Clinically suspicious	Diseased	Error variance	*p*
	*N* = 83	*N* = 74	*N* = 30		
C14:0	1.70 ± 0.08	1.62 ± 0.08	1.66 ± 0.07	0.1474	0.417
C15:0	0.11 ± 0.02	0.09 ± 0.02	0.10 ± 0.02	0.0075	0.281
C16:0	27.1 ± 0.31	27.2 ± 0.32	27.0 ± 0.27	2.1208	0.693
C16:1	3.34 ± 0.24	3.29 ± 0.24	3.28 ± 0.20	1.2502	0.946
C17:0	0.35 ± 0.02	0.35 ± 0.02	0.35 ± 0.02	0.0103	0.928
C18:0	6.44^b^ ± 0.23	6.84^a^ ± 0.18	6.76^ab^ ± 0.15	0.7152	0.011
C18:1n9t	0.15 ± 0.02	0.16 ± 0.02	0.12 ± 0.02	0.0086	0.150
C18:1n9c	34.51 ± 3.18	35.44 ± 2.98	35.20 ± 0.56	9.3735	0.153
C18:2n6c	21.66^a^ ± 0.56	20.52^b^ ± 0.55	20.99^ab^ ± 0.48	6.7727	0.025
C20:0	0.11 ± 0.02	0.13 ± 0.02	0.11 ± 0.01	0.0066	0.532
C18:3n6	0.28 ± 0.02	0.29 ± 0.03	0.25 ± 0.02	0.0135	0.365
C20:1	0.31 ± 0.02	0.30 ± 0.02	0.35 ± 0.02	0.0098	0.140
C18:3n3	1.44^a^ ± 0.05	1.32^b^ ± 0.05	1.40^ab^ ± 0.04	0.0530	0.006
C20:2	0.61^ab^ ± 0.01	0.59^b^ ± 0.02	0.63^b^ ± 0.01	0.0049	0.039
C22:0	0.031 ± 0.01	0.034 ± 0.01	0.027 ± 0.01	0.0026	0.817
C20:3n6	0.28 ± 0.02	0.25 ± 0.02	0.25 ± 0.02	0.0101	0.120
C22:1n9	0 ± 0.002	0.003 ± 0.002	0.002 ± 0.002	0.0001	0.192
C20:3n3	0.065 ± 0.01	0.055 ± 0.01	0.068 ± 0.01	0.0047	0.564
C20:4n6	1.14 ± 0.04	1.13 ± 0.04	1.13 ± 0.03	0.0360	0.894
C22:2	0.019 ± 0.01	0.014 ± 0.01	0.018 ± 0.01	0.0013	0.757
C24:0	0.20 ± 0.02	0.21 ± 0.02	0.21 ± 0.02	0.0101	0.795
C20:5n3	0.019 ± 0.01	0.017 ± 0.01	0.021 ± 0.01	0.0015	0.844
C24:1	0.127^a^ ± 0.02	0.101^a^ ± 0.02	0.085^b^ ± 0.01	0.0066	0.029
C22:6n3	0.027 ± 0.01	0.019 ± 0.01	0.016 ± 0.01	0.0027	0.482
n6:n3 ratio	15.2^b^ ± 0.33	15.9^a^ ± 0.34	15.2^b^ ± 0.29	2.4504	0.021

^a,b,c^ Values within a row with different superscripts differ significantly at *p* < 0.05.

**Table 3:** Comparison of contents of 24 different fatty acids, crude protein and total solids in colostrum of sows in different parity groups (parity group 1–4). Data in g/kg whole colostrum (LSMean ± SE).

Item	Parity 1 + 2	Parity 3 + 4	Parity 5 + 6	Parity ≥ 7	Error variance	*p*
	*N* = 93	*N* = 109	*N* = 61	*N* = 71		
C14:0	0.61/a ± 0.04	0.60^a^ ± 0.04	0.50^b^ ± 0.04	0.51^b^ ± 0.03	0.0603	0.006
C15:0	0.05^ab^ ± 0.01	0.06^a^ ± 0.01	0.04^bc^ ± 0.01	0.03^c^ ± 0.01	0.0014	< 0.0001
C16:0	10.59^a^ ± 0.65	10.40^a^ ± 0.63	8.21^b^ ± 0.72	8.03^b^ ± 0.49	17.0033	< 0.0001
C16:1	1.12 ± 0.09	1.20 ± 0.08	0.98 ± 0.09	1.09 ± 0.06	0.2911	0.098
C17:0	0.147^a^ ± 0.01	0.143^a^ ± 0.01	0.105^b^ ± 0.01	0.094^b^ ± 0.01	0.0038	< 0.0001
C18:0	2.68^a^ ± 0.17	2.56^a^ ± 0.17	2.04^b^ ± 0.19	1.90^b^ ± 0.13	1.1713	< 0.0001
C18:1n9t	0.07^a^ ± 0.01	0.07^a^ ± 0.01	0.05^b^ ± 0.01	0.04^b^ ± 0.01	0.0018	< 0.0001
C18:1n9c	13.29^a^ ± 0.88	13.22^a^ ± 0.85	10.63^b^ ± 0.98	10.69^b^ ± 0.66	31.2164	0.001
C18:2n6c	7.81^a^ ± 0.53	8.06^a^ ± 0.51	6.57^b^ ± 0.59	6.39^b^ ± 0.40	11.2626	0.002
C20:0	0.06^a^ ± 0.01	0.060^ab^ ± 0.01	0.04^bc^ ± 0.01	0.03^c^ ± 0.01	0.0014	< 0.0001
C18:3n6	0.11^ab^ ± 0.01	0.12^a^ ± 0.01	0.09^b^ ± 0.01	0.09^b^ ± 0.01	0.0035	0.009
C20:1	0.11 ± 0.01	0.12 ± 0.01	0.10 ± 0.01	0.11 ± 0.01	0.0033	0.173
C18:3n3	0.53^a^ ± 0.04	0.54^a^ ± 0.03	0.44^b^ ± 0.04	0.43^b^ ± 0.03	0.0510	0.002
C20:2	0.21 ± 0.01	0.23 ± 0.01	0.19 ± 0.01	0.19 ± 0.01	0.0083	0.031
C22:0	0.03^a^ ± 0.004	0.02^a^ ± 0.004	0.01^b^ ± 0.005	0.01^b^ ± 0.003	0.0008	< 0.0001
C20:3n6	0.10^a^ ± 0.01	0.11^a^ ± 0.01	0.09^b^ ± 0.01	0.09^b^ ± 0.01	0.0026	0.014
C22:1n9	0.0020 ± 0.001	0.0004 ± 0.001	0.0007 ± 0.001	0 ± 0	0.0001	0.304
C20:3n3	0.034 ± 0.005	0.034 ± 0.005	0.026 ± 0.006	0.024 ± 0.004	0.0011	0.102
C20:4n6	0.457^a^ ± 0.03	0.426^a^ ± 0.03	0.352^b^ ± 0.03	0.342^b^ ± 0.02	0.0302	< 0.0001
C22:2	0.012 ± 0.003	0.010 ± 0.003	0.007 ± 0.003	0.007 ± 0.002	0.0004	0.394
C24:0	0.086^a^ ± 0.005	0.077^a^ ± 0.005	0.062^b^ ± 0.006	0.058^b^ ± 0.004	0.0012	< 0.0001
C20:5n3	0.016^a^ ± 0.004	0.013^ab^ ± 0.003	0.004^c^ ± 0.004	0.006^bc^ ± 0.003	0.0005	0.003
C24:1	0.044 ± 0.005	0.051 ± 0.005	0.042 ± 0.006	0.039 ± 0.004	0.0011	0.063
C22:6n3	0.027^a^ ± 0.004	0.012^b^ ± 0.004	0.003^c^ ± 0.004	0.002^c^ ± 0.003	0.0006	< 0.0001
Total fatty acids	38.19^a^ ± 2.42	38.11^a^ ± 2.34	30.59^b^ ± 2.68	30.19^b^ ± 1.82	235.24	0.0002
Crude protein	146 ± 5.04	144 ± 4.88	146 ± 5.58	144 ± 3.79	1022.03	0.949
Total solids	224 ± 5.51	224 ± 5.34	217 ± 6.08	214 ± 4.13	1212.05	0.187
n‐6:n‐3 ratio	14.6^b^ ± 0.26	15.1^ab^ ± 0.25	15.4^a^ ± 0.29	15.5^a^ ± 0.20	2.7264	0.004

a,b,c Values within a row with different superscripts differ significantly at *p* < 0.05.

## 4 Results

The proportion of C16:0, C18:1n9c and C18:2n6c among the total fatty acids in colostrum averaged 83.1% ± 0.9%. The analyses of this study also showed that there was no C4:0, C6:0, C8:0, C10:0, C11:0 or C12:0 in the colostrum.

### 4.1 Relationship Between the Health of a Sow and Fatty Acid, Crude Protein and Total Solids Content of Colostrum

The healthy group contained 83 sows (thereof: parity group 1 [*n* = 11]; parity group 2 [*n* = 31]; parity group 3 [*n* = 21]; parity group 4 [*n* = 16]; unknown [*n* = 4]), the clinically suspicious group contained 74 sows (thereof: parity group 1 [*n* = 21]; parity group 2 [*n* = 25]; parity group 3 [*n* = 8]; parity group 4 [*n* = 12]; unknown [*n* = 8]) and the diseased group contained 30 sows (thereof: parity group 1 [*n* = 7]; parity group 2 [*n* = 9]; parity group 3 [*n* = 4]; parity group 4 [*n* = 8]; unknown [*n* = 2]). When comparing the contents of the different fatty acids in % of total fatty acids, the content of C18:0 in the colostrum of clinically suspicious sows was higher than in the colostrum of healthy sows (*p* < 0.05, Table 2). The colostrum of healthy sows had a higher content of C18:2n6c and C18:3n3 than the colostrum of clinically suspicious sows (*p* < 0.05, Table 2). Furthermore, the colostrum of diseased sows had a higher proportion of C20:2 than that of clinically suspicious sows (*p* < 0.05, Table 2). With regard to C24:1, the colostrum of healthy sows had a higher proportion than that of diseased sows (*p* < 0.05, Table 2). The n‐6:n3 ratio was highest in clinically suspicious sows (*p* < 0.05, Table 2).

### 4.2 Influence of the Parity Group of a Sow on Fatty Acid, Crude Protein and Total Solids Content of Colostrum

More significant differences were calculated when sows were not classified according to health but divided into parity groups. The colostrum contents, especially the content of fatty acids in the colostrum of sows of different parity groups, were compared (Table 3). It could be shown that colostrum from sows in parity group 1 and parity group 2 contained more total fatty acids, in particular C14:0, C16:0, C17:0, C18:0, C18:1n9t, C18:1n9c, C18:2n6c, C18:3n3, C22:0, C20:4n6, C24:0 and C22:6n3 than the colostrum of sows in parity group 3 and parity group 4 (*p* < 0.05, Table 3) when measured in g/kg whole colostrum. Regarding C15:0 and C20:0, the colostrum of sows in parity group 1 and parity group 2 showed higher contents than the colostrum of sows in parity group 4 (*p* < 0.05, Table 3). For C18:3n6, the colostrum of sows in parity group 2 indicated a higher content than the colostrum of sows in parity group 3 and 4 (*p* < 0.05, Table 3). Regarding C20:5n3, the colostrum of sows in parity group 1 showed a higher proportion than the colostrum of sows in parity group 3 and 4 (*p* < 0.05, Table 3). The n‐6:n‐3 ratio was lowest in the colostrum of sows in parity group 1.

When comparing the contents of the different parameters in % of total fatty acids, it could be shown that the colostrum of sows in parity group 1 had a higher value for C15:0, C16:0, C17:0, C18:0, C18:1n9t, C20:0, C22:0, C24:0 and C22:6n3 than the colostrum of sows in parity group 4 (*p* < 0.05, Table 4). For C16:1, C18:2n6c and C20:1, the colostrum of sows in parity group 1 showed lower values than the colostrum of sows in parity group 4 (*p* < 0.05, Table 4).

**Table 4:** Comparison of contents of 24 different fatty acids in colostrum of sows of different parity groups (parity group 1–4). Data in % of total fatty acids (LSMean ± SE).

Item	Parity 1 + 2	Parity 3 + 4	Parity 5 + 6	Parity ≥ 7	Error variance	*p*
	*N* = 93	*N* = 109	*N* = 61	*N* = 71		
C14:0	1.66 ± 0.06	1.63 ± 0.06	1.68 ± 0.06	1.73 ± 0.04	0.1384	0.400
C15:0	0.11^a^ ± 0.01	0.13^a^ ± 0.01	0.10^ab^ ± 0.01	0.07^b^ ± 0.01	0.0072	0.0002
C16:0	27.96^a^ ± 0.22	27.34^b^ ± 0.22	26.86^bc^ ± 0.25	26.60^c^ ± 0.17	2.0095	< 0.0001
C16:1	3.07^b^ ± 0.17	3.25^ab^ ± 0.16	3.33^ab^ ± 0.18	3.64^a^ ± 0.12	1.0980	0.007
C17:0	0.39^a^ ± 0.02	0.37^ab^ ± 0.02	0.34^b^ ± 0.02	0.30^c^ ± 0.01	0.0111	< 0.0001
C18:0	7.06^a^ ± 0.14	6.71^b^ ± 0.14	6.63^bc^ ± 0.15	6.34^c^ ± 0.11	0.7846	< 0.0001
C18:1n9t	0.17^a^ ± 0.01	0.17^ab^ ± 0.01	0.13^bc^ ± 0.02	0.12^c^ ± 0.01	0.0088	0.0003
C18:1n9c	34.71 ± 0.47	34.55 ± 0.45	34.92 ± 0.52	35.34 ± 0.35	8.8806	0.360
C18:2n6c	20.20^b^ ± 0.42	21.19^a^ ± 0.41	21.37^a^ ± 0.46	21.24^a^ ± 0.32	7.0658	0.015
C20:0	0.15^a^ ± 0.01	0.13^ab^ ± 0.01	0.11^bc^ ± 0.01	0.09^c^ ± 0.01	0.0065	< 0.0001
C18:3n6	0.28 ± 0.02	0.30 ± 0.02	0.29 ± 0.02	0.29 ± 0.01	0.0128	0.541
C20:1	0.27^c^ ± 0.02	0.30^bc^ ± 0.01	0.33^ab^ ± 0.02	0.35^a^ ± 0.01	0.0096	< 0.0001
C18:3n3	1.38 ± 0.04	1.42 ± 0.04	1.45 ± 0.04	1.42 ± 0.03	0.0601	0.346
C20:2	0.56^c^ ± 0.01	0.60^b^ ± 0.01	0.63^a^ ± 0.01	0.64^a^ ± 0.01	0.0047	< 0.0001
C22:0	0.057^a^ ± 0.01	0.047^ab^ ± 0.01	0.029^bc^ ± 0.01	0.014^c^ ± 0.01	0.0031	< 0.0001
C20:3n6	0.26 ± 0.02	0.26 ± 0.02	0.26 ± 0.02	0.27 ± 0.01	0.0103	0.747
C22:1n9	0.003 ± 0.002	0.001 ± 0.002	0.001 ± 0.002	0 ± 0.001	0.0001	0.322
C20:3n3	0.067 ± 0.01	0.070 ± 0.01	0.061 ± 0.01	0.058 ± 0.01	0.0048	0.667
C20:4n6	1.21^a^ ± 0.03	1.13^b^ ± 0.03	1.15^ab^ ± 0.03	1.16^ab^ ± 0.02	0.0333	0.021
C22:2	0.020 ± 0.01	0.017 ± 0.01	0.015 ± 0.01	0.014 ± 0.01	0.0015	0.780
C24:0	0.24^a^ ± 0.02	0.20^b^ ± 0.01	0.19^b^ ± 0.02	0.20^b^ ± 0.01	0.0092	0.004
C20:5n3	0.027^a^ ± 0.01	0.024^a^ ± 0.01	0.008^b^ ± 0.01	0.013^ab^ ± 0.01	0.0016	0.010
C24:1	0.098 ± 0.01	0.120 ± 0.01	0.114 ± 0.01	0.111 ± 0.01	0.0068	0.288
C22:6n3	0.054^a^ ± 0.01	0.024^b^ ± 0.01	0.007^bc^ ± 0.01	0.004^c^ ± 0.01	0.0026	< 0.0001

a,b,c Values within a row with different superscripts differ significantly at *p* < 0.05.

### 4.3 Interaction Between Health With Regard to PDS and Parity Group

Interaction between health and parity were shown for C14:0 and C16:0 when comparing data in per cent of total fatty acids (*p* < 0.05, Tables 5 and 7). Interaction between health and parity were shown for C18:0, C18:1n9c, C20:0, C18:3n6, C20:1, C20:3n3, C20:4n6 and C22:6n3 when comparing data in g/kg whole colostrum (*p* < 0.05, Tables 6 and 8).

**Table 5:** Results of the two‐way ANOVA test considering interaction effects of health status (healthy, clinically suspicious and diseased) and parity (parity group 1–4), comparing data in % of total fatty acids.

Item	Health effect			Parity effect			Interaction effect		
	*N* = 172			*N* = 172			*N* = 172		
	df	*F*	*p*	df	*F*	*p*	df	*F*	*p*
C14:0	2	0.05	0.951	3	2.23	0.087	6	2.72	0.015
C15:0	2	1.38	0.254	3	3.73	0.013	6	0.69	0.660
C16:0	2	0.52	0.598	3	9.09	< 0.001	6	3.15	0.006
C16:1	2	0.12	0.885	3	1.47	0.225	6	1.67	0.133
C17:0	2	0.20	0.816	3	3.30	0.022	6	1.78	0.106
C18:0	2	1.21	0.300	3	2.01	0.115	6	1.66	0.133
C18:1n9t	2	2.19	0.116	3	4.69	0.004	6	0.20	0.975
C18:1n9c	2	0.77	0.463	3	1.44	0.234	6	1.37	0.229
C18:2n6c	2	1.49	0.228	3	0.63	0.598	6	0.35	0.909
C20:0	2	0.23	0.792	3	5.13	0.002	6	2.08	0.059
C18:3n6	2	1.27	0.282	3	1.28	0.282	6	0.73	0.628
C20:1	2	2.88	0.059	3	3.92	0.010	6	2.07	0.060
C18:3n3	2	2.08	0.129	3	0.28	0.841	6	1.30	0.261
C20:2	2	2.66	0.073	3	12.63	< 0.001	6	1.08	0.376
C22:0	2	0.58	0.563	3	4.03	0.009	6	1.28	0.269
C20:3n6	2	2.77	0.066	3	0.73	0.535	6	1.40	0.219
C22:1n9	2	3.05	0.050	3	0.56	0.644	6	0.67	0.676
C20:3n3	2	2.03	0.135	3	1.99	0.118	6	2.03	0.065
C20:4n6	2	0.18	0.836	3	2.17	0.093	6	1.35	0.239
C22:2	2	0.75	0.475	3	0.30	0.824	6	1.28	0.268
C24:0	2	0.35	0.709	3	3.39	0.019	6	0.88	0.514
C20:5n3	2	0.74	0.478	3	2.29	0.080	6	0.74	0.614
C24:1	2	5.03	0.008	3	3.44	0.018	6	1.60	0.150
C22:6n3	2	2.66	0.073	3	6.79	< 0.001	6	1.50	0.183
n‐6:n‐3 ratio	2	4.28	0.016	3	3.87	0.011	6	0.28	0.946

**Table 6:** Results of the two‐way ANOVA test considering interaction effects of health status (healthy, clinically suspicious and diseased) and parity (parity group 1–4) comparing data in g/kg whole colostrum.

Item	Health effect			Parity effect			Interaction effect		
	*N* = 172			*N* = 172			*N* = 172		
	df	*F*	*p*	df	*F*	*p*	df	*F*	*p*
C14:0	2	2.38	0.010	3	3.43	0.019	6	0.78	0.588
C15:0	2	1.78	0.173	3	4.64	0.004	6	1.10	0.363
C16:0	2	1.50	0.226	3	5.71	0.001	6	2.19	0.046
C16:1	2	0.94	0.393	3	2.36	0.073	6	0.70	0.654
C17:0	2	1.13	0.324	3	6.42	< 0.001	6	1.99	0.07
C18:0	2	0.63	0.534	3	6.24	< 0.001	6	3.44	0.003
C18:1n9t	2	1.78	0.171	3	6.09	< 0.001	6	1.04	0.398
C18:1n9c	2	0.95	0.388	3	3.88	0.010	6	3.01	0.008
C18:2n6c	2	2.21	0.113	3	4.55	0.004	6	2.15	0.050
C20:0	2	0.68	0.509	3	6.63	< 0.001	6	2.58	0.021
C18:3n6	2	1.39	0.253	3	3.99	< 0.001	6	2.37	0.032
C20:1	2	0.36	0.700	3	3.75	0.012	6	3.00	0.008
C18:3n3	2	2.88	0.059	3	3.78	0.012	6	1.39	0.221
C20:2	2	1.24	0.293	3	4.00	0.009	6	3.01	0.008
C22:0	2	0.49	0.611	3	4.03	0.009	6	1.38	0.225
C20:3n6	2	3.03	0.051	3	3.32	0.021	6	2.14	0.052
C22:1n9	2	2.60	0.077	3	0.37	0.774	6	0.44	0.853
C20:3n3	2	1.49	0.229	3	2.49	0.062	6	2.69	0.016
C20:4n6	2	2.28	0.106	3	4.65	0.004	6	2.40	0.030
C22:2	2	0.47	0.624	3	0.36	0.784	6	1.29	0.264
C24:0	2	1.39	0.253	3	6.87	< 0.001	6	1.02	0.414
C20:5n3	2	0.75	0.474	3	2.08	0.105	6	1.05	0.394
C24:1	2	4.00	0.020	3	4.78	0.003	6	1.97	0.074
C22:6n3	2	2.98	0.054	3	7.09	< 0.001	6	2.39	0.031
Total fatty acids	2	1.44	0.240	3	4.79	0.003	6	2.56	0.021

## 5 Discussion

Colostrum quality plays a particularly important role in ensuring piglet growth, health and survival (Hasan et al. 2019; Bondoc et al. 2020; Roszkos et al. 2020; Blavi et al. 2021). Despite a higher n‐6:n‐3 ratio in clinically suspicious sows, this study found no significant differences between sows’ health status regarding PDS and other analysed colostrum components, including total solids and fatty acids. This may be attributable to the fact that the mammary glands were often healthy while PDS‐infection. The same indications were also provided by a previous study (Kaiser et al. 2020).

Our study and previous studies reported that the proportion of fatty acids with the highest amounts in colostrum (C16:0, C18:1n9c and C18:2n6c) among the total fatty acids is about 80%, ranging from 79.5% to 83.5% (Csapó et al. 1996; Aguinaga et al. 2016; Luise et al. 2018). Some fatty acids were not found in the colostrum (C4:0, C6:0, C8:0, C10:0, C11:0 and C12:0). These findings are in accordance with previous studies (Csapó et al. 1996; Lauridsen and Danielsen 2004) that did not find these fatty acids in colostrum either. However, another study was able to demonstrate that the content of C12:0 in sow milk is strongly influenced by the content of C12:0 in the sow's diet (Świątkiewicz et al. 2020). In addition, a previous study found C8:0, C10:0 and C12:0 in colostrum (Settachaimongkon et al. 2023). The laboratory methodology was similar to that of this study (Settachaimongkon et al. 2023). Therefore, all fatty acids were detected at that site that we would also have identified if they had been present in the colostrum in our study. However, the previous study did not specify what the sows were fed. Therefore, differences in feed are the most plausible explanation for the detection of C8:0, C10:0 and C12:0 in the colostrum in the previous study (Settachaimongkon et al. 2023).

### 5.1 Relationship Between the Health of a Sow and Fatty Acid, Crude Protein and Total Solids Content of Colostrum

The physiological state of the mammary gland is an important determinant in the composition of mammary secretions. The data in percentage of total fatty acids revealed that the percentage of C18:0 in the colostrum of clinically suspicious sows was higher than in the colostrum of sows without PDS. A previous study suggested that high proportions of C18:0 in colostrum and milk have a negative effect on the daily weight gain of piglets and increase the preweaning mortality of piglets (Skrzypczak et al. 2015). However, daily weight gain and mortality of piglets did not differ between piglets of healthy (without PDS) and clinically suspicious sows in our investigations (Rosengart et al. 2021; Rosengart et al. 2022). The colostrum of healthy sows had a higher proportion of C18:2n6c as well as C18:3n3 than the colostrum of clinically suspicious sows. To our knowledge, this has not been reported in the literature on pigs. Furthermore, the colostrum of PDS‐diseased sows had a higher proportion of C20:2 compared with that of clinically suspicious sows. It is not yet possible to estimate whether and, if so, what consequences this may have. With regard to C24:1, the colostrum of PDS‐diseased sows had a lower percentage than that of healthy sows. Colostrum synthesis begins approximately 10 days before farrowing (Palombo et al. 2018). However, this appears unlikely as an explanatory hypothesis, as most constituents described below are similar between the compared sow groups. To our knowledge, levels of C24:1 in the colostrum of sows have not yet been studied in relation to health status regarding PDS. In humans, C24:1 is found primarily in the central nervous system (Kageyama et al. 2021). Moreover, C24:1 seems to have a positive effect on liver inflammation in a mouse model of Parkinson's disease (Wang et al. 2023). Until now, the impact of C24:1 on the development and health of suckling piglets has not been assessed. Furthermore, the n‐6:n‐3 ratio in the colostrum of clinically suspicious sows in our study was higher than in the colostrum of healthy and PDS‐diseased sows. We are unable to explain this result and could not find any indications in the literature either. Every other result of our present study showed no relation between the health status regarding PDS of individual sows and the examined composition of their colostrum, as no significant differences could be observed regarding total solids and fatty acids based on g/kg whole colostrum and total solids, respectively. Crude protein levels did not differ according to health status regarding PDS in this study, either. The different components of the crude protein in colostrum are important for the transfer of immunity (immunoglobulins), stimulation of growth (growth factors) and facilitation of fat digestion (enzymes), but not for supplying energy. The primary protein components of colostrum are immunoglobulins, including the IgG, IgA and IgM (Hurley 2003). During the first few hours after parturition, lactating sows give colostrum characterised by a very high concentration of protein, that is, immunoglobulins, and rather relatively low fat and lactose concentration levels on the first day of lactation (Csapó et al. 1996; Aguinaga et al. 2016; Curtasu et al. 2016). All in all, the health status regarding PDS of the sows had little influence on the percentage of examined fatty acids in the colostrum. Colostrum synthesis begins approximately 10 days before farrowing and concludes a few hours after farrowing (Palombo et al. 2018; Quesnel and Farmer 2019). Most analysed constituents of colostrum were similar between the compared groups of sows. Therefore, it appears unlikely that PDS had already developed within the 10 days prior to parturition to an extent that it could have influenced colostrum production. However, the low number of fatty acids that showed differences may serve as potential early biomarkers for the impending onset of PDS. In addition, a possible reason for the similarity of most constituents could be that PDS often occurred without clinical mastitis (Kaiser et al. 2020). It must also be taken into account that a PDS disease often restricts feed intake only for a very short time. Therefore, the dietary impact of PDS often might be low. Another possible reason might be that colostrum sampling did not always take place at the same time as the clinical examination of the sows. Further insights could be provided by studies in which clinical examination and colostrum sampling are conducted simultaneously. Nevertheless, interactions between health status regarding PDS and parity group with regard to single fatty acids were observed, which are discussed in the section on the interaction between health status regarding PDS and parity. Currently, not much is known from the literature about the relation between sow health status regarding PDS in the late gestation phase and shortly after farrowing and the colostrum components. This justifies the study approach of comparing colostrum content and health status regarding PDS shortly after farrowing (Tables 7 and 8).

**Table 7:** Least square mean (LSMean) ± standard error (SE) for each interaction effect of health status (healthy, clinically suspicious and diseased) × parity group (parity group 1–4). Data in % of total fatty acid.

	HxP1	HxP2	HxP3	HxP4	CSxP1	CSxP2	CSxP3	CSxP4	DxP1	DxP2	DxP3	DxP4
C14:0	1.54 ± 0.18	1.72 ± 0.15	1.62 ± 0.17	1.74 ± 0.16	1.70 ± 0.16	1.48 ± 0.15	1.70 ± 0.19	1.70 ± 0.17	2.10 ± 0.20	1.44 ± 0.18	1.52 ± 0.23	1.56 ± 0.13
C15:0	0.11 ± 0.04	0.14 ± 0.03	0.08 ± 0.04	0.07 ± 0.03	0.10 ± 0.03	0.11 ± 0.03	0.06 ± 0.04	0.03 ± 0.04	0.07 ± 0.04	0.11 ± 0.04	0.10 ± 0.05	0.10 ± 0.03
C16:0	27.09 ± 0.61	27.57 ± 0.52	27.08 ± 0.59	26.27 ± 0.55	28.14 ± 0.55	26.78 ± 0.54	26.69 ± 0.66	27.09 ± 0.60	28.59 ± 0.68	27.19 ± 0.64	25.97 ± 0.81	25.74 ± 0.47
C16:1	2.68 ± 0.52	3.32 ± 0.45	3.24 ± 0.50	3.73 ± 0.47	3.03 ± 0.47	3.11 ± 0.46	3.26 ± 0.56	3.97 ± 0.51	4.02 ± 0.58	2.92 ± 0.55	3.01 ± 0.69	3.15 ± 0.40
C17:0	0.38 ± 0.05	0.38 ± 0.04	0.31 ± 0.04	0.30 ± 0.04	0.39 ± 0.04	0.36 ± 0.04	0.37 ± 0.05	0.26 ± 0.04	0.34 ± 0.05	0.33 ± 0.05	0.40 ± 0.06	0.35 ± 0.03
C18:0	7.05 ± 0.38	6.37 ± 0.33	6.81 ± 0.36	6.03 ± 0.34	7.02 ± 0.34	6.99 ± 0.33	6.52 ± 0.41	6.60 ± 0.37	6.69 ± 0.42	6.99 ± 0.40	6.64 ± 0.50	6.75 ± 0.29
C18:1n9t	0.17 ± 0.04	0.17 ± 0.04	0.13 ± 0.04	0.12 ± 0.04	0.16 ± 0.04	0.17 ± 0.04	0.10 ± 0.05	0.11 ± 0.04	0.12 ± 0.05	0.16 ± 0.04	0.06 ± 0.06	0.09 ± 0.03
C18:1n9c	35.53 ± 1.40	33.65 ± 1.19	34.85 ± 1.33	35.39 ± 1.24	34.49 ± 1.25	35.75 ± 1.22	36.07 ± 1.50	35.72 ± 1.37	33.34 ± 1.56	35.48 ± 1.46	37.03 ± 1.84	35.73 ± 1.06
C18:2n6c	20.69 ± 1.22	21.99 ± 1.04	21.33 ± 1.17	21.79 ± 1.09	20.36 ± 1.09	20.70 ± 1.07	21.19 ± 1.32	20.25 ± 1.20	20.36 ± 1.36	20.86 ± 1.28	21.00 ± 1.61	21.66 ± 0.93
C20:0	0.13 ± 0.04	0.12 ± 0.03	0.12 ± 0.03	0.08 ± 0.03	0.16 ± 0.03	0.14 ± 0.03	0.04 ± 0.04	0.08 ± 0.04	0.11 ± 0.04	0.15 ± 0.04	0.04 ± 0.05	0.12 ± 0.03
C18:3n6	0.30 ± 0.06	0.28 ± 0.05	0.27 ± 0.05	0.29 ± 0.05	0.28 ± 0.05	0.32 ± 0.05	0.26 ± 0.06	0.24 ± 0.06	0.24 ± 0.06	0.28 ± 0.06	0.17 ± 0.07	0.27 ± 0.04
C20:1	0.28 ± 0.05	0.28 ± 0.04	0.29 ± 0.04	0.36 ± 0.04	0.28 ± 0.04	0.32 ± 0.04	0.27 ± 0.05	0.31 ± 0.04	0.24 ± 0.05	0.36 ± 0.05	0.43 ± 0.06	0.37 ± 0.03
C18:3n3	1.32 ± 0.10	1.48 ± 0.09	1.41 ± 0.10	1.42 ± 0.09	1.36 ± 0.09	1.29 ± 0.09	1.37 ± 0.11	1.30 ± 0.10	1.46 ± 0.12	1.30 ± 0.11	1.45 ± 0.14	1.40 ± 0.08
C20:2	0.57 ± 0.03	0.59 ± 0.03	0.64 ± 0.03	0.64 ± 0.03	0.56 ± 0.03	0.61 ± 0.03	0.59 ± 0.03	0.63 ± 0.03	0.55 ± 0.03	0.62 ± 0.03	0.67 ± 0.04	0.69 ± 0.02
C22:0	0.05 ± 0.02	0.04 ± 0.02	0.04 ± 0.02	0.01 ± 0.02	0.04 ± 0.02	0.06 ± 0.02	0.01 ± 0.02	0 ± 0.02	0.04 ± 0.03	0.03 ± 0.02	0 ± 0.03	0.02 ± 0.02
C20:3n6	0.29 ± 0.05	0.28 ± 0.04	0.24 ± 0.05	0.27 ± 0.04	0.27 ± 0.04	0.25 ± 0.04	0.15 ± 0.05	0.23 ± 0.05	0.20 ± 0.05	0.24 ± 0.05	0.28 ± 0.06	0.28 ± 0.04
C22:1n9	0 ± 0.004	0 ± 0.004	0 ± 0.004	0 ± 0.004	0.003 ± 0.004	0.002 ± 0.004	0.01 ± 0.004	0 ± 0.004	0 ± 0.005	0 ± 0.004	0 ± 0.005	0 ± 0.003
C20:3n3	0.09 ± 0.03	0.06 ± 0.03	0.06 ± 0.03	0.07 ± 0.03	0.06 ± 0.03	0.08 ± 0.03	0.01 ± 0.03	0.03 ± 0.03	0.03 ± 0.03	0.09 ± 0.03	0.03 ± 0.04	0.09 ± 0.02
C20:4n6	1.20 ± 0.09	1.15 ± 0.08	1.15 ± 0.08	1.08 ± 0.08	1.20 ± 0.08	1.09 ± 0.08	1.10 ± 0.09	1.13 ± 0.09	1.18 ± 0.10	1.02 ± 0.09	1.06 ± 0.12	1.23 ± 0.07
C22:2	0.02 ± 0.02	0.02 ± 0.01	0.02 ± 0.02	0.02 ± 0.02	0.02 ± 0.02	0.02 ± 0.01	0.01 ± 0.02	0 ± 0.02	0 ± 0.02	0.02 ± 0.02	0 ± 0.02	0.04 ± 0.01
C24:0	0.23 ± 0.05	0.22 ± 0.04	0.16 ± 0.04	0.18 ± 0.04	0.25 ± 0.04	0.19 ± 0.04	0.19 ± 0.05	0.22 ± 0.05	0.26 ± 0.05	0.20 ± 0.05	0.12 ± 0.06	0.24 ± 0.04
C20:5n3	0.04 ± 0.02	0.02 ± 0.02	0.01 ± 0.02	0.01 ± 0.02	0.03 ± 0.02	0.02 ± 0.02	0 ± 0.02	0 ± 0.02	0.02 ± 0.02	0.02 ± 0.02	0 ± 0.02	0.03 ± 0.01
C24:1	0.13 ± 0.04	0.13 ± 0.03	0.13 ± 0.04	0.11 ± 0.03	0.09 ± 0.03	0.13 ± 0.03	0.04 ± 0.04	0.11 ± 0.04	0.04 ± 0.04	0.13 ± 0.04	0.03 ± 0.05	0.10 ± 0.03
C22:6n3	0.09 ± 0.02	0.02 ± 0.02	0.02 ± 0.02	0.01 ± 0.02	0.05 ± 0.02	0.01 ± 0.02	0 ± 0.02	0 ± 0.02	0.02 ± 0.03	0.04 ± 0.02	0 ± 0.03	0 ± 0.02

Abbreviations: H, healthy regarding postpartum dysgalactia syndrome (PDS); CS, clinically suspicious regarding PDS; D, diseased regarding PDS; P1, parity group 1; P2, parity group 2; P3, parity group 3; P4, parity group 4.

**Table 8:** Least square mean (LSMean) ± standard error (SE) for each interaction effect of health status (healthy, clinically suspicious and diseased) × parity group (parity group 1–4). Data in g/kg whole colostrum.

	HxP1	HxP2	HxP3	HxP4	CSxP1	CSxP2	CSxP3	CSxP4	DxP1	DxP2	DxP3	DxP4
C14:0	0.68 ± 0.11	0.60 ± 0.10	0.52 ± 0.11	0.58 ± 0.10	0.62 ± 0.10	0.60 ± 0.10	0.38 ± 0.12	0.42 ± 0.11	0.51 ± 0.13	0.60 ± 0.12	0.36 ± 0.15	0.53 ± 0.09
C15:0	0.06 ± 0.02	0.05 ± 0.01	0.03 ± 0.02	0.03 ± 0.01	0.05 ± 0.01	0.05 ± 0.01	0.02 ± 0.02	0.01 ± 0.02	0.03 ± 0.02	0.05 ± 0.02	0.03 ± 0.02	0.04 ± 0.01
C16:0	12.15 ± 1.87	9.68 ± 1.60	8.66 ± 1.79	8.66 ± 1.66	10.68 ± 1.67	11.24 ± 1.64	6.49 ± 2.01	6.74 ± 1.84	7.27 ± 2.09	11.66 ± 1.96	6.08 ± 2.47	8.98 ± 1.42
C16:1	1.19 ± 0.26	1.14 ± 0.22	1.00 ± 0.25	1.25 ± 0.23	1.14 ± 0.23	1.28 ± 0.23	0.78 ± 0.28	1.01 ± 0.25	0.89 ± 0.29	1.20 ± 0.27	0.69 ± 0.34	1.14 ± 0.20
C17:0	0.17 ± 0.03	0.14 ± 0.02	0.10 ± 0.03	0.10 ± 0.02	0.14 ± 0.02	0.15 ± 0.02	0.08 ± 0.03	0.07 ± 0.03	0.09 ± 0.03	0.14 ± 0.03	0.09 ± 0.03	0.12 ± 0.02
C18:0	3.15 ± 0.48	2.23 ± 0.41	2.12 ± 0.45	2.01 ± 0.42	2.66 ± 0.43	2.95 ± 0.42	1.64 ± 0.51	1.63 ± 0.47	1.70 ± 0.53	3.02 ± 0.50	1.57 ± 0.63	2.30 ± 0.36
C18:1n9t	0.08 ± 0.02	0.06 ± 0.02	0.05 ± 0.02	0.05 ± 0.02	0.07 ± 0.02	0.07 ± 0.02	0.03 ± 0.02	0.03 ± 0.02	0.04 ± 0.02	0.07 ± 0.02	0.02 ± 0.02	0.04 ± 0.01
C18:1n9c	16.09 ± 2.59	11.85 ± 2.21	10.92 ± 2.47	12.01 ± 2.30	13.10 ± 2.31	15.17 ± 2.26	9.01 ± 2.78	8.91 ± 2.54	8.37 ± 2.88	15.31 ± 2.71	8.49 ± 3.41	12.87 ± 1.97
C18:2n6c	9.17 ± 1.48	7.76 ± 1.26	6.81 ± 1.41	7.29 ± 1.31	7.73 ± 1.32	8.65 ± 1.29	5.28 ± 1.59	4.91 ± 1.45	5.41 ± 1.65	8.91 ± 1.54	4.84 ± 1.95	7.54 ± 1.12
C20:0	0.07 ± 0.02	0.05 ± 0.01	0.05 ± 0.01	0.04 ± 0.01	0.06 ± 0.01	0.06 ± 0.01	0.02 ± 0.02	0.02 ± 0.02	0.03 ± 0.02	0.07 ± 0.02	0.02 ± 0.02	0.05 ± 0.01
C18:3n6	0.14 ± 0.03	0.11 ± 0.02	0.09 ± 0.03	0.10 ± 0.02	0.11 ± 0.02	0.13 ± 0.02	0.07 ± 0.03	0.07 ± 0.03	0.06 ± 0.03	0.13 ± 0.03	0.05 ± 0.04	0.11 ± 0.02
C20:1	0.13 ± 0.03	0.10 ± 0.02	0.10 ± 0.03	0.12 ± 0.02	0.11 ± 0.02	0.14 ± 0.02	0.08 ± 0.03	0.09 ± 0.03	0.07 ± 0.03	0.16 ± 0.03	0.09 ± 0.04	0.14 ± 0.02
C18:3n3	0.58 ± 0.09	0.52 ± 0.08	0.45 ± 0.09	0.48 ± 0.08	0.51 ± 0.08	0.53 ± 0.08	0.33 ± 0.10	0.31 ± 0.09	0.38 ± 0.11	0.55 ± 0.10	0.34 ± 0.13	0.48 ± 0.07
C20:2	0.25 ± 0.04	0.21 ± 0.04	0.20 ± 0.04	0.21 ± 0.04	0.21 ± 0.04	0.26 ± 0.04	0.15 ± 0.05	0.15 ± 0.04	0.14 ± 0.05	0.27 ± 0.04	0.15 ± 0.06	0.24 ± 0.03
C22:0	0.03 ± 0.01	0.02 ± 0.01	0.02 ± 0.01	0.01 ± 0.01	0.02 ± 0.01	0.03 ± 0.01	0.01 ± 0.01	0 ± 0.01	0.02 ± 0.01	0.02 ± 0.01	0 ± 0.01	0.01 ± 0.01
C20:3n6	0.13 ± 0.02	0.10 ± 0.02	0.09 ± 0.02	0.09 ± 0.02	0.10 ± 0.02	0.11 ± 0.02	0.05 ± 0.03	0.06 ± 0.02	0.06 ± 0.03	0.11 ± 0.02	0.06 ± 0.03	0.11 ± 0.02
C22:1n9	0 ± 0.003	0 ± 0.003	0 ± 0.003	0 ± 0.003	0.01 ± 0.003	0 ± 0.003	0 ± 0.003	0 ± 0.003	0 ± 0.003	0 ± 0.003	0 ± 0.004	0 ± 0.002
C20:3n3	0.05 ± 0.01	0.03 ± 0.01	0.03 ± 0.01	0.03 ± 0.01	0.03 ± 0.01	0.04 ± 0.01	0.01 ± 0.02	0.01 ± 0.01	0.01 ± 0.02	0.05 ± 0.02	0.01 ± 0.02	0.04 ± 0.01
C20:4n6	0.54 ± 0.08	0.40 ± 0.07	0.37 ± 0.07	0.36 ± 0.07	0.44 ± 0.07	0.46 ± 0.07	0.27 ± 0.08	0.28 ± 0.08	0.31 ± 0.09	0.43 ± 0.08	0.25 ± 0.10	0.41 ± 0.06
C22:2	0.01 ± 0.01	0.01 ± 0.01	0.01 ± 0.01	0.01 ± 0.01	0.01 ± 0.01	0.01 ± 0.01	0.01 ± 0.01	0 ± 0.01	0 ± 0.01	0.01 ± 0.01	0 ± 0.01	0.02 ± 0.01
C24:0	0.09 ± 0.02	0.08 ± 0.01	0.06 ± 0.01	0.06 ± 0.01	0.09 ± 0.01	0.08 ± 0.01	0.05 ± 0.02	0.05 ± 0.01	0.07 ± 0.02	0.08 ± 0.02	0.03 ± 0.02	0.07 ± 0.01
C20:5n3	0.03 ± 0.01	0.01 ± 0.01	0 ± 0.01	0.01 ± 0.01	0.01 ± 0.01	0.01 ± 0.01	0 ± 0.01	0 ± 0.01	0.01 ± 0.01	0.01 ± 0.01	0 ± 0.01	0.02 ± 0.01
C24:1	0.06 ± 0.02	0.05 ± 0.01	0.05 ± 0.01	0.04 ± 0.01	0.04 ± 0.01	0.06 ± 0.01	0.02 ± 0.02	0.03 ± 0.01	0.02 ± 0.02	0.06 ± 0.02	0.01 ± 0.02	0.04 ± 0.01
C22:6n3	0.05 ± 0.01	0.01 ± 0.01	0.01 ± 0.01	0.01 ± 0.01	0.02 ± 0.01	0.01 ± 0.01	0 ± 0.01	0 ± 0.01	0.01 ± 0.01	0.02 ± 0.01	0 ± 0.01	0 ± 0.01
Total fatty acids	44.87 ± 6.96	35.21 ± 5.94	31.74 ± 6.64	33.74 ± 6.19	37.98 ± 6.23	42.10 ± 6.09	24.78 ± 7.49	24.81 ± 6.84	25.48 ± 7.76	42.92 ± 7.28	23.18 ± 9.18	35.29 ± 5.30

Abbreviations: H, healthy regarding postpartum dysgalactia syndrome (PDS); CS, clinically suspicious regarding PDS; D, diseased regarding PDS; P1, parity group 1; P2, parity group 2; P3, parity group 3; P4, parity group 4.

### 5.2 Influence of the Parity Group of a Sow on Fatty Acid, Crude Protein and Total Solids Content of Colostrum

It is known that parity can influence swine colostrum and milk composition, as it has been reported in previous studies (Declerck et al. 2015; Bondoc et al. 2020; Luise et al. 2020; Segura et al. 2020; Amatucci et al. 2022).

In the present study, the results confirmed the effect of parity on some fatty acids, as the sows in parity group 1 and parity group 2 showed a greater amount of total fatty acids when compared to the sows in parity group 3 and parity group 4.

Nuntapaitoon et al. (2020) reported that the sow parity number had no impact on examined colostrum composition in Danish Landrace × Yorkshire crossbred sows in Thailand. They compared the content of fat, protein and lactose in colostrum from 27 gilts, 48 sows from parity 2–4 and 30 sows from parity 5 to 6. However, in agreement with the present study, other studies reported that the concentration of total fatty acids in the colostrum is affected or tends to be affected by the parity of the sow, with a higher concentration in primiparous sows (Declerck et al. 2015; Segura et al. 2020; Amatucci et al. 2022). Two recent studies reported high fat levels in the colostrum of gilts and decreasing fat levels in the colostrum of older sows (Segura et al. 2020; Amatucci et al. 2022). In an older study, Mahan (1998) reported a linear decrease in colostral fat content as parity advanced, with the largest decline occurring from parity 1 to parity 2. Nevertheless, the results of the study by Mahan and Peters (2004) noted that parity does not appear to affect fat percentage in sow colostrum, at least up to parity 4. That is in agreement with the result in our study. Perhaps genetic differences between sows investigated by Mahan (1998) and Mahan and Peters (2004) explain those different results. Both those studies are characterised by a relatively large sample size. Nevertheless, the sample size of all previously mentioned studies was much smaller than that in the present study. However, it should be mentioned that we combined two parities to form a parity group, which was necessary to achieve a sufficient sample size.

The n‐6:n‐3 ratio was the lowest in the colostrum of sows in parity group 1. Nguyen et al. (2020) reported improved weaning survival rates and weight gain of suckling piglets and an increased amount of total n‐3 fatty acids in colostrum when sows were fed a diet with a low n‐6:n‐3 ratio. The improved weaning survival rates and the weight gain of suckling piglets could be influenced by the promotion of intestinal health by inhibiting toll‐like receptor 4 and a positive modulation of the intestinal microbiome through more n‐3 fatty acids (Chen et al. 2019). Another study reported about heavier piglets 3 days after weaning when sows were fed a diet with a low n‐6:n‐3 ratio in comparison to sows fed a diet with a higher n‐6:n‐3 ratio (McAfee et al. 2019). The three aforementioned studies agree that a low n‐6:n‐3 ratio in colostrum and milk is beneficial for the piglets. In this respect, the colostrum of younger sows seems to be of a higher quality.

In our study, the parity group of the sow did not affect the crude protein content of colostrum. The same results can also be found in the literature (Klobasa et al. 1987; Göransson 1990; Baas et al. 1992) and are confirmed by a previous study examining Danish hybrid sows (Landrace × Yorkshire) kept in the tropical climate zone (Nuntapaitoon et al. 2020).

The fact that the clinical examination of the sows and the colostrum sampling did not always occur at the same time is certainly a methodological influencing factor, as disease in some cases may have developed after colostrum sampling. The same applies to the fact that the colostrum was not always sampled from the same teat position, as colostrum composition may vary between anterior and posterior teats. Methodologically, it would have been better to conduct sampling and clinical examination at the same time and to take colostrum from the same teat position. However, due to work‐related reasons, this was not possible, and a great advantage of our dataset is its very large sample size.

Storage temperature and storage length for samples may have influenced our results. We stored the colostrum samples at −20°C. The majority of studies did the same (Yao et al. 2012; Krogh et al. 2015; Skrzypczak et al. 2015; Aguinaga et al. 2016; Bai et al. 2017), but a few other studies stored the colostrum samples at −80°C (Luise et al. 2018; Amatucci et al. 2022). None of the previous studies mentioned the colostrum storage duration.

Regarding the fatty acid composition, a few differences were observed, but demonstrating the significance of these differences is challenging. We have to emphasise that we did not measure the colostrum yield; however, a previous study showed no differences in colostrum yield for sows across the first nine parities (Quesnel 2011). Another study showed that colostrum yield tended to be highest in parity 2 and parity 3 sows (Devillers et al. 2007). If that is true, piglets from older sows (parity ≥ 5) are generally less well supplied with various fatty acids (C14:0, C15:0, C16:0, C17:0, C18:0, C18:1n9t, C18:1n9c, C18:2n6c, C20:0, C18:3n6, C18:3n3, C22:0, C20:3n6, C20:4n6, C24:0, C20:5n3 and C22:6n3) from colostrum compared to those from younger sows (parity ≤ 4). If older sows have the same colostrum yield as younger sows (or a lower yield) and at the same time a lower content of special fatty acids, it might make sense to ensure older sows receive a better supply of fatty acids in their feed as a precaution. Several previous studies have shown that an increased fat level in feed also increases the fat level and fatty acid profile in colostrum and milk (Shurson et al. 1986; Leonard et al. 2010; Laws et al. 2018; Nguyen et al. 2020; Vodolazska and Lauridsen 2020). Feeding on the farm was highly standardised during the peripartum period. Thus, each sow, including gilts, received approximately the same amount of feed. For this reason, the differences in the content of individual fatty acids according to parity cannot be explained by differences in the amount of feed consumed by the sows according to their parity.

### 5.3 Interaction Between Health With Regard to PDS and Parity Group

Significant interactions between health status and parity group were observed for selected fatty acids, indicating that parity‐related effects depend on the animal's health condition with regard to PDS (Tables 5, 6, 7, 8). When expressed as a percentage of total fatty acids, interactions were limited to C14:0 and C16:0 (*p* < 0.05). In contrast, expression on a mass basis (g/kg whole colostrum) revealed interaction effects for a broader range of mainly long‐chain fatty acids, including C18:0, C18:1n9c, C20:0, C18:3n6, C20:1, C20:3n3, C20:4n6 and C22:6n3. These findings suggest that metabolic and physiological differences associated with parity groups sometimes could be modulated by health status, maybe reflecting alterations in lipid mobilisation and mammary synthesis with regard to PDS. A previous study showed alterations in serum concentrations of some metabolites regarding parity, implementing differences in lipid mobilisation regarding parity (Gregory and Huber 2025). The more often pronounced interactions in absolute concentrations indicate that variation in total lipid output contributes substantially to these effects. In a previous study, while transition phase interaction between health and parity in the milk of dairy cows could be shown for some ingredients, fatty acids were not analysed (Ruprechter et al. 2018). Unfortunately, we can't find more references to these findings in the literature, especially for sows.

## 6 Conclusion

In summary, our results suggest that the health status regarding PDS of the sows shortly after farrowing has almost no link to the examined fatty acid composition of the colostrum. Nevertheless, it must be emphasised that there are single interactions between health status regarding PDS and parity group that we can't explain conclusively. In addition, the data showed that the parity group of the sows affected the examined composition of the colostrum, especially with regard to individual fatty acids. More research is needed to better understand the relationship between colostrum content and piglet health and to better understand the causes of the interactions mentioned above.

## Author Contributions

Conceptualisation: Stephan Rosengart, Michael Wendt and Christian Visscher. Methodology: Stephan Rosengart, Michael Wendt and Christian Visscher. Validation: Stephan Rosengart, Florian Lohkamp, Hubert Henne, Anne Kathrin Appel, Lea‐Sophie Trost, Jens Tetens, Imke Traulsen, Ansgar Deermann, Michael Wendt and Christian Visscher. Formal analysis: Stephan Rosengart, Florian Lohkamp and Christian Visscher. Data curation: Stephan Rosengart. Writing – original draft preparation: Stephan Rosengart, Florian Lohkamp and Bussarakam Chuppava. Writing – review and editing: Bussarakam Chuppava, Hubert Henne, Anne Kathrin Appel, Lea‐Sophie Trost, Jens Tetens, Imke Traulsen, Ansgar Deermann, Michael Wendt and Christian Visscher. Visualisation: Stephan Rosengart and Christian Visscher. Supervision: Bussarakam Chuppava, Michael Wendt and Christian Visscher. Funding acquisition: Hubert Henne, Anne Kathrin Appel, Imke Traulsen, Jens Tetens, Ansgar Deermann, Michael Wendt and Christian Visscher. All authors have read and agreed to the published version of the manuscript.

## Ethics Statement

Sampling and animal housing were performed in accordance with the German rules and regulations and were approved by the Animal Welfare Officer of the University of Veterinary Medicine Hannover, Foundation, Hannover, Germany (reference: TVO‐2020‐V‐9).

## Conflicts of Interest

Ansgar Deermann is employed by EVH Select GmbH, Meppen, Germany and VzF GmbH, Uelzen, Germany. Anne Kathrin Appel and Hubert Henne are employed by BHZP GmbH, Dahlenburg‐Ellringen, Germany. The remaining authors declare that the research was conducted in the absence of any commercial or financial relationships that could be construed as a potential conflict of interest.

## Supporting information


Supporting File

## Data Availability

None of the data were deposited in the official repository. Data will be available upon request (contact stephan.rosengart@tiho-hannover.de).
