# Supplementary material for: An Investigation of Fatty Acids, Crude Protein and Total Solids in Colostrum in Relation to Health Status Regarding Postpartum Dysgalactia Syndrome and Certain Parity Groups of Sows
Source: J Anim Physiol Anim Nutr (Berl). 2026 Aug 6;110(6):918–33. doi: 10.1111/jpn.70100 (PMC13768916; doi:10.1111/jpn.70100)
Supplement: Supplementary file 1 [file JPN-110-918-s001.docx]

An investigation of the colostrum composition in relation to the health status and parity of sows

S. Rosengart, F. Lohkamp, B. Chuppava, H. Henne, A.-K. Appel, L.-S. Trost, J. Tetens, I. Traulsen, A. Deermann, M. Wendt, C. Visscher

**Supplementary material**

**Supplementary Table S1.**

Different nutrient contents including 25 fatty acids in the gestation and lactation diet (pelleted complete feed) in accordance with the analysis. Data in g/kg of total solids.

|  | Gestation Diet | Lactation Diet |
| --- | --- | --- |
| Item | N=4 | N=4 |
| Crude protein | 158 ± 3.83 | 182 ± 11.3 |
| Crude fat | 33.5 ± 1.68 | 40.3 ± 4.42 |
| Crude fibre | 80.6 ± 6.53 | 53.3 ± 4.10 |
| Crude ash | 59.1 ± 1.56 | 58.0 ± 3.33 |
| Calcium | 8.01 ± 1.08 | 8.98 ± 1.07 |
| Phosphorus | 5.39 ± 0.42 | 6.71 ± 0.26 |
| Energy (MJ ME/kg) | 13.2 ± 0.29 | 14.6 ± 0.23 |
| C12:0 | 0.1 ± 0 | <0.05 |
| C14:0 | 0.09 ± 0.02 | 0.06 ± 0.01 |
| C15:0 | <0.05 | <0.05 |
| C16:0 | 6.77 ± 0.11 | 6.79 ± 0.38 |
| C16:1 | 0.07 ± 0.01 | 0.06 ± 0.01 |
| C17:0 | <0.05 | <0.05 |
| C18:0 | 0.56 ± 0.01 | 0.78 ± 0.04 |
| C18:1n9t | <0.05 | <0.05 |
| C18:1n9c | 5.21 ± 0.44 | 5.12 ± 0.40 |
| C18:2n6c | 16.96 ± 0.60 | 20.68 ± 1.21 |
| C20:0 | 0.08 ± 0.001 | 0.08 ± 0.01 |
| C18:3n6 | <0.05 | <0.05 |
| C20:1 | 0.19 ± 0.01 | 0.17 ± 0.02 |
| C18:3n3 | 1.36 ± 0.07 | 2.03 ± 0.14 |
| C20:2 | 0.05 ± 0.0002 | 0.05 ± 0.002 |
| C22:0 | 0.10 ± 0.003 | 0.10 ± 0.004 |
| C20:3n6 | <0.05 | <0.05 |
| C22:1n9 | <0.05 | <0.05 |
| C20:3n3 | <0.05 | <0.05 |
| C20:4n6 | <0.05 | <0.05 |
| C22:2 | <0.05 | <0.05 |
| C24:0 | 0.11 ± 0.02 | 0.09 ± 0.01 |
| C20:5n3 | <0.05 | <0.05 |
| C24:1 | 0.07 ± 0.003 | 0.05 ± 0 |
| C22:6n3 | <0.05 | <0.05 |
| Total fatty acids | 31.64 ± 0.82 | 36.04 ± 2.14 |
| n-6:n-3 ratio | 12.46 ± 0.36 | 10.18 ± 0.48 |

**Supplementary Table S2**

Sow’s daily intake of feed and analysed feed components in g/day, depending on the expected farrowing date. Day 0 is the calculated date of farrowing (115th day of gestation).

|  | Distance to calculated farrowing date in days | | | | |
| --- | --- | --- | --- | --- | --- |
| Item | -7 to -3 | -2 | -1 | 0 | 1 + 2 |
| Gestation Diet | 3311 | 1656 | 993 | 331 | - |
| Lactation Diet | - | - | 665 | 1331 | 3326 |
| Crude Protein | 523 | 261 | 278 | 294 | 605 |
| Crude fat | 111 | 55.5 | 60.1 | 64.7 | 134 |
| Crude fibre | 267 | 133 | 116 | 98 | 177 |
| Crude ash | 196 | 97.8 | 97.3 | 96.7 | 193 |
| Calcium | 265 | 133 | 139 | 146 | 299 |
| Phosphorus | 178 | 89.2 | 98.2 | 107 | 223 |
| Energy (MJ ME) | 43.7 | 21.9 | 22.8 | 23.8 | 48.6 |
| C12:0 | 0.331 | 0.166 | 0.099 | 0.033 | <0.05 |
| C14:0 | 0.298 | 0.149 | 0.129 | 0.110 | 0.200 |
| C15:0 | <0.05 | <0.05 | <0.05 | <0.05 | <0.05 |
| C16:0 | 22.4 | 11,2 | 11.2 | 11.3 | 22.6 |
| C16:1 | 0.231 | 0.116 | 0.109 | 0.103 | 0.200 |
| C17:0 | <0.05 | <0.05 | <0.05 | <0.05 | <0.05 |
| C18:0 | 1.85 | 0.927 | 1.08 | 1.22 | 2.59 |
| C18:1n9t | <0.05 | <0.05 | <0.05 | <0.05 | <0.05 |
| C18:1n9c | 17.3 | 8.63 | 8.58 | 8.54 | 17.0 |
| C18:2n6c | 56.2 | 28.1 | 30.6 | 33.1 | 68.8 |
| C20:0 | 0.265 | 0.132 | 0.133 | 0.133 | 0.266 |
| C18:3n6 | <0.05 | <0.05 | <0.05 | <0.05 | <0.05 |
| C20:1 | 0.629 | 0.315 | 0.302 | 0.289 | 0.565 |
| C18:3n3 | 4.50 | 2.25 | 2.70 | 3.15 | 6.75 |
| C20:2 | 0.166 | 0.083 | 0.083 | 0.083 | 0.166 |
| C22:0 | 0.331 | 0.166 | 0.166 | 0.166 | 0.333 |
| C20:3n6 | <0.05 | <0.05 | <0.05 | <0.05 | <0.05 |
| C22:1n9 | <0.05 | <0.05 | <0.05 | <0.05 | <0.05 |
| C20:3n3 | <0.05 | <0.05 | <0.05 | <0.05 | <0.05 |
| C20:4n6 | <0.05 | <0.05 | <0.05 | <0.05 | <0.05 |
| C22:2 | <0.05 | <0.05 | <0.05 | <0.05 | <0.05 |
| C24:0 | 0.364 | 0.182 | 0.169 | 0.156 | 0.299 |
| C20:5n3 | <0.05 | <0.05 | <0.05 | <0.05 | <0.05 |
| C24:1 | 0.232 | 0.116 | 0.103 | 0.090 | 0.166 |
| C22:6n3 | <0.05 | <0.05 | <0.05 | <0.05 | <0.05 |
| Total fatty acids | 104 | 52.4 | 55.4 | 58.4 | 120 |

**Supplementary Table S3.**

Standard deviation, variance and coefficient of variance as measures of analytical precision.

| Item | Standard deviation in g/kg | Variance (Standard deviation2) | Coefficient of variance in % of identified item |
| --- | --- | --- | --- |
| C14:0 | 0.02295 | 0.00053 | 0.57 |
| C15:0 | 0.02236 | 0.00050 | 1.11 |
| C16:0 | 0.05423 | 0.00294 | 0.90 |
| C16:1 | 0.02947 | 0.00087 | 1.46 |
| C17:0 | 0.02845 | 0.00081 | 1.41 |
| C18:0 | 0.06366 | 0.00405 | 1.60 |
| C18:1n9t | 0.02865 | 0.00082 | 1.42 |
| C18:1n9c | 0.05876 | 0.00345 | 1.47 |
| C18:2n6c | 0.04346 | 0.00189 | 2.15 |
| C20:0 | 0.06092 | 0.00371 | 1.51 |
| C18:3n6 | 0.03234 | 0.00105 | 1.59 |
| C20:1 | 0.03788 | 0.00144 | 1.89 |
| C18:3n3 | 0.01642 | 0.00027 | 0.82 |
| C20:2 | 0.03707 | 0.00137 | 1.85 |
| C22:0 | 0.07120 | 0.00507 | 1.77 |
| C20:3n6 | 0.02446 | 0.00060 | 1.22 |
| C22:1n9 | 0.03777 | 0.00143 | 1.88 |
| C20:3n3 | 0.02722 | 0.00074 | 1.35 |
| C20:4n6 | 0.04614 | 0.00213 | 2.31 |
| C22:2 | 0.03738 | 0.00140 | 1.87 |
| C24:0 | 0.06943 | 0.00482 | 1.74 |
| C20:5n3 | 0.02850 | 0.00081 | 1.42 |
| C24:1 | 0.04334 | 0.00188 | 2.16 |
| C22:6n3 | 0.03519 | 0.00124 | 1.76 |
| Crude protein | 2.71 | 7.34 | 1.70 |
